# *Borrelia turicatae* from Ticks in Peridomestic Setting, Camayeca, Mexico

**DOI:** 10.3201/eid3002.231053

**Published:** 2024-02

**Authors:** Edwin Vázquez-Guerrero, Alexander R. Kneubehl, Patricio Pellegrini-Hernández, José Luis González-Quiroz, María Lilia Domínguez-López, Aparna Krishnavajhala, Paulina Estrada-de los Santos, J. Antonio Ibarra, Job E. Lopez

**Affiliations:** Instituto Politécnico Nacional, Mexico City, Mexico (E. Vázquez-Guerrero, J.L. González-Quiroz, M.L. Domínguez-López, P. Estrada-de los Santos, J.A. Ibarra);; Baylor College of Medicine, Houston, Texas, USA (A.R. Kneubehl, A. Krishnavajhala, J.E. Lopez);; Wildlife Conservation Management Unit (Macochín), El Fuerte, Sinaloa, Mexico (P. Pellegrini-Hernández).

**Keywords:** *Borrelia turicatae*, ticks, Mexico, tickborne diseases, vector-borne infections, bacteria

## Abstract

We conducted surveillance studies in Sinaloa, Mexico, to determine the circulation of tick-borne relapsing fever spirochetes. We collected argasid ticks from a home in the village of Camayeca and isolated spirochetes. Genomic analysis indicated that *Borrelia turicatae* infection is a threat to those living in resource-limited settings.

Tick-borne relapsing fever (TBRF) spirochetes are neglected pathogens in Mexico, and human infection is frequently misdiagnosed because of nonspecific symptoms (*1*). *Borrelia turicatae* infection is associated with irregular fevers, vomiting, rigors, nausea, and meningitis (*2*). The neurologic symptoms that follow infection can be misdiagnosed as Lyme disease, and the use of nonspecific serologic tests further complicates an accurate diagnosis of TBRF. Prior studies have used whole-protein lysates of *Borreliella* (*Borrelia*) *burgdorferi* in ELISAs and immunoblotting assays for disease diagnosis (*3*,*4*), but serologic cross-reactivity occurs regardless of whether patients are infected with Lyme-causing or TBRF-causing spirochetes (*5*). Another report from Mexico amplified a portion of the *flagellin* gene from a patient’s blood sample, and it most closely aligned with *B. burgdorferi* (*6*). However, no other loci were sequenced, and it is unknown if the isolate causing infection exists. Additional work is needed to understand the circulation of spirochetes in Mexico.

Argasid ticks transmit most species of TBRF spirochetes, and the life cycle of those ticks further confounds a clear understanding of the disease’s epidemiology. Argasids in the genus *Ornithodoros* are cavity-dwelling rapid feeders that are rarely found attached to the host. A case study from Panama noted persons who reported being bitten by insects during their sleep (*7*). An investigation identified *Ornithodoros puertoricensis* ticks under floor tiles and within cracks of windowsills (*7*). Those findings indicated that, once introduced into the dwelling, the ticks targeted human occupants as their primary blood source.

To clarify the ecologic overlap of TBRF spirochetes and humans, we initiated efforts to collect argasid ticks from peridomestic settings in Mexico. We describe identification of *Ornithodoros turicata* ticks from the village of Camayeca in Sinaloa, Mexico. We determined infection in the collected ticks by feeding them on a laboratory mouse, isolating TBRF spirochetes from the mouse blood, and confirming the pathogen as *B. turicatae* through genomic analysis*.*

## The Study

**Figure 1:**
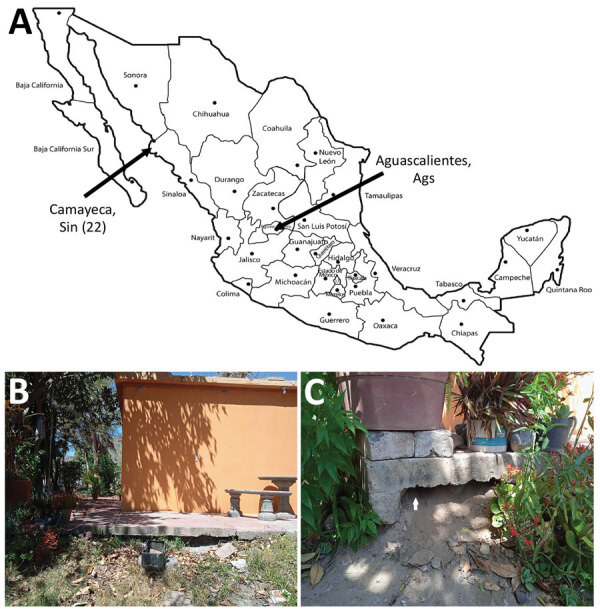
Collection of *Ornithodoros turicata* ticks from village of Camayeca, Mexico. A) Location in the state of Sinaloa where 22 ticks were collected (left arrow). Also labeled is the state of Aguascalientes, where we recently collected *O. turicata* ticks (right arrow). B) Collection efforts were focused in peridomestic settings C) Ticks were aspirated from the base of a human dwelling; white arrow indicates where ticks were collected.

In March 2022, we collected argasid ticks in peridomestic settings of Sinaloa, Mexico. In the village of Camayeca (Figure 1, panel A), we sampled 5 burrows using an aspirator or dry ice as a source of carbon dioxide to lure ticks. In the human dwelling where ticks were collected (Figure 1, panel B), we aspirated the dirt at the base of the home (Figure 1, panel C). We collected 3 adults and 19 nymphs. We also noted ground squirrel activity around the burrows.

In the laboratory, we speciated ticks using microscopy and by sequencing a portion of the 16S mitochondrial gene. Morphologic characterization of nymphs and adults identified them as *O. turicata.* We extracted total DNA from 3 nymphs using the DNeasy Blood and Tissue kit (QIAGEN, https://www.qiagen.com), according to the manufacturer’s protocol, and amplified ≈475 nt of the 16S mitochondrial rRNA gene by using Tm16S+1 and Tm16S-1 primers (*8*). We sequenced amplicons by using the Sanger method and trimmed the data by using ChromasPro version 2.1.5 (Technelysium Pty Ltd, https://technelysium.com.au). We performed a BLASTN analysis (https://www.ncbi.nlm.nih.gov/Blast.cgi), which indicated 99.1% nucleotide identity to *O. turicata.* We deposited sequences from this study into GenBank (accession no. OR189376–8).

**Figure 2:**
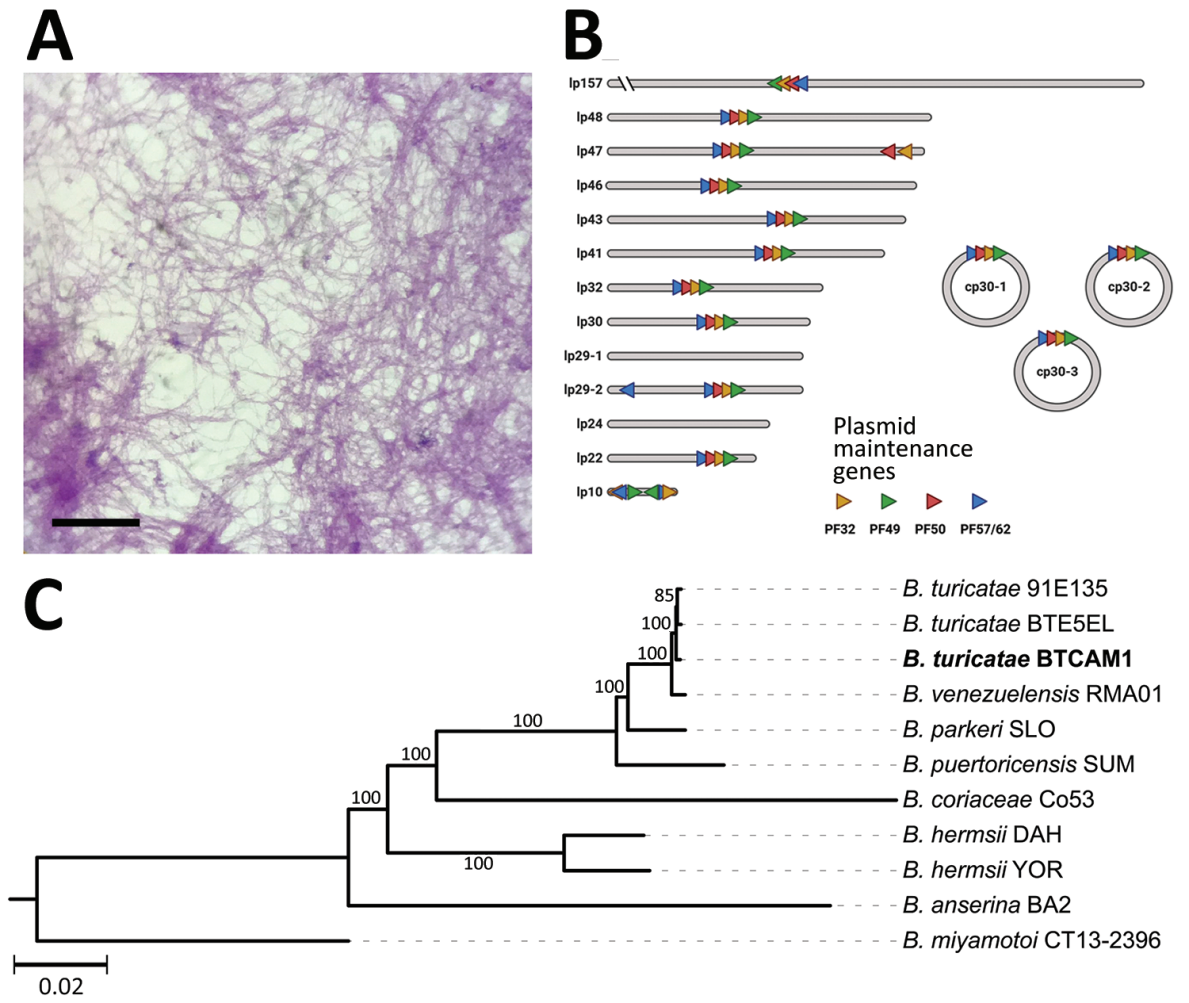
Isolation and genetic characterization of *Borrelia turicatae* from ticks collected in the village of Camayeca, Mexico. A) Spirochetes were isolated from murine blood in culture medium. Scale bar indicates 20 µm. B) Genome sequencing and assembly generated the plasmid repertoire of the bacteria. Plasmids were designated as lp or cp and by their respective size to the nearest kilobase. PF partitioning genes are shown in each plasmid as orange, green, red, and blue triangles. C) Maximum-likelihood species tree performed in a phylogenomic analysis of the spirochete sample we extracted, designated CAM-1 (boldface), grouped the spirochete with *B. turicatae.* The tree was generated with an edge-linked proportional partition model with 1,000 ultra-fast bootstraps. Scale bar indicates 0.02 substitutions per site. cp, circular plasmid; lp, linear plasmid; PF, plasmid family.

We did not evaluate the remaining 19 individual *O. turicata* ticks for infection because we did not want to sacrifice them; however, we determined colonization of TBRF spirochetes by allowing them to feed on a BALB/c mouse and then assessing the animal for infection. We collected daily blood samples from the mouse and performed Giemsa staining to visualize spirochetes. Seven days after feeding ticks, we exsanguinated the mouse and centrifuged whole blood at 500 × *g* for 5 minutes. We then removed plasma and centrifuged again at 5,000 × *g* for 10 minutes. We resuspended the resulting pellet in 1 mL of Barbour-Stoenner-Kelly–IIB media and cultured in a total of 4 mL of the media formulation at 35°C (*9*). Eight days later, we placed an aliquot of the culture on a glass slide, allowed it to air dry, and performed Giemsa staining. We visualized numerous spirochetes on the slide (Figure 2, panel A). We designated the isolate CAM-1 and generated glycerol stocks.

We sequenced the CAM-1 isolate to determine the species and genomic structure. We isolated genomic DNA and performed pulsed field electrophoresis to determine DNA quality (*10*). We performed long-read sequencing with the Oxford Nanopore Technologies (https://nanoporetech.com) Mk1B platform with the SQK-RBK110.96 library preparation kit and R9.4.1 flow cell. We generated short-read sequences by using the Microbial Genome Sequencing Center (MiGS Center, https://migscenter.wordpress.com) and an Illumina 2x150 library preparation kit (Illumina, https://www.illumina.com). We produced a plasmid-resolved genome assembly by using short-reads to polish the long-read data, as done previously (*10*). The mean Oxford Nanopore Technologies coverage was 439×, and the mean Illumina coverage was 236×. Using a previously established approach (*10*), we determined a completeness score of 99.89% and a QV score (based on the Phred scale) of Q53.82. We annotated the assembly with the National Center for Biotechnology Information’s Prokaryotic Genome Annotation Pipeline and submitted the assembly to GenBank (accession nos. CP129306–22). The chromosome was 925,885 bp. We noted 16 plasmids, ranging from 10,351 to 156,755 bp, 3 of which were circular (Figure 2, panel B). We used concatenated sequences from 650 core genes in our phylogenomic analysis, which encompassed 720,532 nt and grouped the CAM-1 isolate with *B. turicatae* (Figure 2, panel C).

## Conclusions

*O. turicata* ticks were originally described in Mexico in 1876 by Alfredo Dugès (*11*) but were implicated as a vector of TBRF spirochetes until the 1930s. In 1933, Brumpt et al. detected spirochetes in *O. turicata* ticks collected from Austin, Texas, USA, and he later confirmed that the tick transmitted *B. turicatae* (*12*). At the same time, Pilz and Mooser observed human cases of relapsing fever in the city of Aguascalientes, Mexico (*13*). Their work showed that *O. turicata* ticks were in the region, and the researchers implicated that species as the vector. Since those reports, studies from Mexico on *Ornithodoros* ticks ecology or TBRF spirochetes have been negligible.

Our findings indicate that updates are needed for distribution models of *O. turicata* ticks*.* For example, a maximum entropy species distribution model predicted suitable habitat for *O. turicata* ticks by using georeferenced data points from tick collections and reports of *B. turicatae*, primarily from the United States (*14*). New regions of northern Mexico were predicted to have habitat for *O. turicata* ticks, but there was low probability of suitable habitat in other areas of the country. However, in addition to collecting ticks from Camayeca, Mexico, we also have recovered *O. turicata* ticks from the city of Aguascalientes, Mexico (Figure 1, panel A) (*15*). The 2 cities are >1,000 km apart. Aguascalientes is located in the middle of the country and is considered a temperate environment, ≈1,900 m in elevation, whereas Camayeca is an arid desert region at ≈150 m elevation. The environmental differences between the 2 cities show wider habitat suitability for *O. turicata* ticks than what was previously predicted.

Identifying infected *O. turicata* ticks in a peridomestic setting suggests that TBRF is likely underreported in Mexico. In support of this finding, retrospective serodiagnostic studies detected human exposure to TBRF spirochetes in populations originally diagnosed with fever of unknown origin (*1*). Given those observations and our findings, additional studies should be conducted to determine infection frequencies of argasid ticks collected in peridomestic settings and to define the distribution and ecology of *O. turicata* ticks and other argasid ticks of human importance in Mexico to increase knowledge and awareness of these ticks and the potential threat they pose to animal and human health.
